# Interferon Alpha Signalling and Its Relevance for the Upregulatory Effect of Transporter Proteins Associated with Antigen Processing (TAP) in Patients with Malignant Melanoma

**DOI:** 10.1371/journal.pone.0146325

**Published:** 2016-01-06

**Authors:** Ruth Heise, Philipp M. Amann, Silke Ensslen, Yvonne Marquardt, Katharina Czaja, Sylvia Joussen, Daniel Beer, Rupert Abele, Gabriele Plewnia, Robert Tampé, Hans F. Merk, Heike M. Hermanns, Jens M. Baron

**Affiliations:** 1 Department of Dermatology and Allergology, RWTH Aachen University, Aachen, Germany; 2 IZKF, RWTH Aachen University, Aachen, Germany; 3 Institute of Biochemistry, Biocenter, Goethe-University Frankfurt, Frankfurt am Main, Germany; 4 Medical Clinic and Policlinic II, Hepatology, University Hospital Würzburg, Würzburg, Germany; University of Alabama at Birmingham, UNITED STATES

## Abstract

**Introduction:**

Interferon alpha (IFNα) is routinely used in the clinical practice for adjuvant systemic melanoma therapy. Understanding the molecular mechanism of IFNα effects and prediction of response in the IFNα therapy regime allows initiation and continuation of IFNα treatment for responder and exclusion of non-responder to avoid therapy inefficacy and side-effects. The transporter protein associated with antigen processing-1 (TAP1) is part of the MHC class I peptide-loading complex, and important for antigen presentation in tumor and antigen presenting cells. In the context of personalized medicine, we address this potential biomarker TAP1 as a target of IFNα signalling.

**Results:**

We could show that IFNα upregulates TAP1 expression in peripheral blood mononuclear cells (PBMCs) of patients with malignant melanoma receiving adjuvant high-dose immunotherapy. IFNα also induced expression of TAP1 in mouse blood and tumor tissue and suppressed the formation of melanoma metastasis in an *in vivo* B16 tumor model. Besides its expression, TAP binding affinity and transport activity is induced by IFNα in human monocytic THP1 cells. Furthermore, our data revealed that IFNα clearly activates phosphorylation of STAT1 and STAT3 in THP1 and A375 melanoma cells. Inhibition of Janus kinases abrogates the IFNα-induced TAP1 expression. These results suggest that the JAK/STAT pathway is a crucial mediator for TAP1 expression elicited by IFNα treatment.

**Conclusion:**

We suppose that silencing of TAP1 expression provides tumor cells with a mechanism to escape cytotoxic T-lymphocyte recognition. The observed benefit of IFNα treatment could be mediated by the shown dual effect of TAP1 upregulation in antigen presenting cells on the one hand, and of TAP1 upregulation in ‘silent’ metastatic melanoma cells on the other hand. In conclusion, this work contributes to a better understanding of the mode of action of IFNα which is essential to identify markers to predict, assess and monitor therapeutic response of IFNα treatment in the future.

## Introduction

Although malignant melanoma accounts for only 4 percent of all dermatologic cancers it is responsible for 80 percent of all deaths from skin cancer [1]. Despite intensive clinical and research efforts during recent decades the prognosis of melanoma patients in advanced tumor stage remains poor [2]. Interferon alpha-2b (IFNα-2b) was shown to affect disease behaviour reproducibly in large randomized controlled clinical trials in an adjuvant setting improving relapse-free survival but its influence on overall survival is still discussed controversially [3–8]. Recently, a large trial showed that also adjuvant ipilimumab significantly and clearly improved recurrence-free survival for patients with completely resected high-risk stage III melanoma [9]. However, in contrast to IFNα therapy, adverse event profile was more severe.

IFNα regulates tumor cell growth and differentiation by affecting cellular communication and signal transduction pathways elicited by this cytokine. Its signalling takes place through the JAK (Janus kinase)/STAT (signal transducers and activators of transcription) pathway. Upon IFNα binding to its cell surface specific receptor the activated receptor-associated Janus kinases (JAKs) JAK1 and TYK2 phosphorylate STAT1 and STAT2 proteins [10]. Activated STAT1 and STAT2 proteins form a heterodimer that associates with IRF9 (interferon regulatory factor 9), resulting in the IFN-stimulated gene factor 3 (ISGF3) complex. After translocation into the nucleus, this complex initiates transcription by binding to conserved IFN-stimulated response element (ISRE) sequence elements within the promoters of IFNα-responsive genes [11,12]. Additionally, activated STAT1 proteins form STAT1/STAT1 homodimers which translocate to the nucleus and initiate gene transcription by binding to the gamma activated sequence (GAS) elements in gene promoters of IFNα-responsive genes [13,14]. Transcription factors of the IFN regulatory factor (IRF) family, such as IRF1, are also induced by this pathway and interact with the specific interferon consensus sequence (ICS) in gene promoters of IFNα responsive genes to induce target gene transcription. Apart from the JAK/STAT pathway the mitogen-activated protein kinase (MAPK) signalling pathways, in particular the ERK and p38 MAPK signal cascade, may also be important for the IFNα dependent biological responses. The p38 MAPK or ERK is rapidly phosphorylated and activated in response to IFNα treatment and is responsible for the transcriptional activation of IFNα target genes [11,15,16]. Furthermore IFNα activates the transcription factor STAT3 [13]. Another important pathway of signal transduction upon IFNα stimulation is one involving the phosphatidyl inositol-3 kinase (PI3K) and its downstream effector Akt [17].

The ATP-binding cassette transporter associated with antigen processing (TAP) belongs to the superfamily of ATP-binding cassette (ABC) transporters, which are found in all kingdoms of life. ABC transporters translocate a very broad spectrum of solutes across biological membranes by binding and hydrolysis of ATP, which is important for a variety of cellular functions [18]. TAP forms a heterodimeric complex consisting of TAP1 and TAP2 and is part of the macromolecular MHC class I peptide-loading complex composed of TAP1, TAP2, tapasin, MHC I heavy chain, β2-microglobulin, and the lectin-like chaperon calreticulin as well as the oxidoreductase ERp57 [19]. As a central part of this peptide-loading complex, TAP plays a key role in the adaptive immune defense against virus-infected or malignantly transformed cells by translocating peptides generated by the proteasome complex from the cytosol into the lumen of the endoplasmatic reticulum (ER), where these peptides are loaded onto MHC class I molecules [19]. These stable peptide-MHC class I complexes traffic via the Golgi compartment to the cell surface where they display their antigenic cargo to CD8^+^-cytotoxic T-lymphocytes, which can thereby efficiently recognize and eliminate infected or malignantly transformed cells [20]. In case of malignant transformation of melanocytes, TAP is involved in the presentation of the tumor specific antigens MART-1 (melanoma antigen recognized by T-cells) or MAGE-1 or -3 (melanoma antigen encoding gene) to cytotoxic T-lymphocytes, leading to an efficient elimination of the malignant cells. Furthermore, a number of molecular mechanisms have been found by which antigenic material is transferred from intact tumor cells to dendritic cells to present tumor specific antigens in a TAP/MHC-I-dependent manner [21–23]. Subsequently, activated cytotoxic T-cells recognize and kill tumor antigen presenting dendritic cells as well as the melanoma cells. Aberrant expression or function of TAP enables tumor cells, including melanoma cells, to escape the immune surveillance by suppression of antigen processing and cell surface presentation of tumor specific peptides via MHC class I molecules which is associated with an ineffective T-cell response [24,25].

The use of an efficient immunotherapy for cancer is focusing on the regeneration of the antigen processing and presentation pathway resulting in T-cell mediated recognition and elimination of tumor antigen presenting cancer and dendritic cells [26]. Although IFNα therapy has been established in the routine treatment of patients with high-risk melanoma [27], the mechanisms of anti-tumor effect and the action of IFNα in adjuvant melanoma therapy still remains unclear. In a first case report, we could detect TAP upregulation in few IFNα-treated patients with stage III malignant melanoma [28] indicating that TAP expression profile could be a possible criteria for the clinical response to interferon therapy [29]. Since Tao et al. (2008) observed that the restoration of TAP function has the potential to enhance melanoma-associated antigen presentation and tumor specific immunity [30] we investigated the role of TAP as a key factor of the adjuvant IFNα immunotherapy for malignant melanoma.

A better understanding of the mode of action could help to predict, assess and monitor therapeutic response of IFNα treatment.

## Materials and Methods

### Ethics statement

All necessary permits were obtained for the described studies. The clinical study in humans was approved by the Ethics committee of the RWTH Aachen University, Aachen, Germany. The participants gave their written informed consent, and the study was conducted according to the principles of the Declaration of Helsinki. The use of mice as animal models was approved by the local authorities of the state of Northrhine Westfalia (Landesamt fuer Natur, Umwelt und Verbraucherschutz NRW, permission number 9.93.2.10.35.07.036), Germany. All experimental procedures causing discomfort of the animals were carried out under general anaesthesia.

### Activators, cytokines and receptors

Recombinant interferon alpha-2b (IntronA, IFNα) was purchased from Essex Pharma GmbH (Munich, Germany). Recombinant human and murine IFNα was obtained from Sigma-Aldrich GmbH (Taufkirchen, Germany). Recombinant IL-6, IL-1β and OSM was purchased from Peprotech (Hamburg, Germany) and sIL-6R was prepared as described before [31]. Lipopolysaccharide from *Escherichia coli* was obtained from Sigma-Aldrich (Taufkirchen, Germany).

### Antibodies

Antibodies against Y701-phosphorylated STAT1 (STAT1-pY701), S727-phosphorylated STAT1 (STAT1-pS727), STAT1, Y705-phosphorylated STAT3 (STAT3-pY705), STAT3, S473-phosphorylated Akt (Akt-pS473), Akt, T202/Y204-phosphorylated ERK1/2 (ERK1pTpY, ERK2pTpY), ERK1/2, tubulin, and the HRP-coupled secondary antibodies were obtained from Cell Signaling Technology (Frankfurt/Main, Germany). The monoclonal antibodies against TAP1 (148.3) was used as described by Meyer et al. (1994) [32] and van Endert et al. (1994) [33].

### Suppliers

Penicillin, streptomycin, sodium pyruvate, L-glutamine and fetal calf serum (FCS) were purchased from Invitrogen-Gibco (Paisley, UK). Geniticin, aprotinin, pepstatin and phenylmethylsulfonyl fluoride (PMSF) were from Sigma-Aldrich (Taufkirchen, Germany). Leupeptin was purchased from MP Biomedicals (Illkirch, France). Sodium orthovanadate was obtained from Merck (Darmstadt, Germany). DMSO and JAK inhibitor I (JI-1) were from Calbiochem (Merck, Darmstadt, Germany). Ficoll Paque and the polyvinylidene difluoride (PVDF) membrane were purchased from GE Healthcare (Munich, Germany). The enhanced chemiluminescence kit was obtained from Thermo Fisher Scientific (Bonn, Germany). Protein concentrations were determined with the Bio-Rad reagent (Bio-Rad, München, Germany).

### Media for cell culture

THP1 cells were grown in RPMI 1640 medium supplemented with 10% heat inactivated FCS, streptomycin (100 mg/l) and penicillin (60 mg/l). A375 cells were grown in RPMI 1640 medium supplemented with 5% FCS, streptomycin (100 mg/l) and penicillin (60 mg/l). B16F1 murine melanoma cells (ATCC, Rockville, MD) were cultured in Dulbecco’s modified eagle medium (DMEM) containing high glucose, L-glutamine and 10% FCS. Peripheral blood mononuclear cells (PBMC) were cultured in RPMI 1640 supplemented with 10% FCS, streptomycin (100 mg/l), penicillin (60 mg/l), and 1% sodium pyruvate (110 mM). All media were obtained from Life Technologies, Invitrogen (Darmstadt, Germany).

### Cell culture

Peripheral blood mononuclear cells (PBMCs) were isolated by density gradient centrifugation using Ficoll Paque as described before [34].

### Patients and IFNα treatment

18 patients diagnosed with UICC (union internationale contre le cancer) stage III malignant melanoma received adjuvant high-dose interferon alpha-2b-treatment (IntronA, IFNα−2b; 20 million IU/m^2^). Before therapy, peripheral blood mononuclear cells (PBMCs) were isolated as control. IFNα was applied intravenously for 5 days per week for 4 weeks according to the Kirkwood protocol [7]. At day 1, 3, 4, 5, 8 and 9, blood samples were taken 2 hours after receiving therapy and PBMCs were isolated immediately.

PBMCs were isolated 4 hours after the last subcutaneous IFNα application (day 19). Total RNA from PBMCs was isolated and subjected to *real time* PCR analysis (qRT-PCR) using specific probes to detect human TAP1 and 18SrRNA as internal reference. Statistical analysis was performed using the *Statistical Analysis System* of the SAS Institute Inc., Cary, NC, USA.

### Cell lysis and Western blotting

Cells were stimulated for the times and with the cytokines and activators indicated in the figure legends. The pharmacological JAK inhibitor I (JI-1) was applied 30 minutes prior to stimulation with cytokines. Immediately after stimulation, cells were lysed in Triton X-100 lysis buffer (20 mM Tris, pH 7.5, 150 mM NaCl, 1% Triton X-100, 1 mM EDTA, 10 mM NaF, 1 mM Na_3_VO_4_, 1 mM PMSF, 3 μg/ml pepstatin, 5 μg/ml aprotinin, and 5 μg/ml leupeptin), as described previously [35,36]. Proteins were separated by sodium dodecyl sulphate (SDS)–polyacrylamide gel electrophoresis in 10% gels, followed by electroblotting onto a PVDF membrane. Antigens were detected by incubation with the indicated specific primary antibodies and horseradish-peroxidase (HRP)-coupled secondary antibodies and developed with an enhanced chemiluminescence kit according to the manufacturer’s instructions. Quantification of the chemiluminescence signal was carried out on the FluorChemQ using the Alpha View^®^ software (ProteinSimple). Equal loading of the gel was verified by stripping the membrane in 62.5 mM Tris HCl (pH 6.7) containing 2% SDS and 100 mM β-mercaptethanol at 70°C for 20 minutes and redetection with antibodies recognizing the protein irrespective of its phosphorylation status as well as by detection of tubulin.

### RNA extraction

Total RNA was isolated from PBMCs of patients treated with IFNα and THP1 and A375 cells stimulated with indicated concentrations of IFNα for the specified time points using the high-pure RNA isolation kit (Roche, Mannheim, Germany) or the RNeasy kit (Qiagen, Hilden, Germany) according to the manufacturers’ instructions. Total RNA from mouse blood was isolated using the PAXgene™ Blood RNA System (PreAnalytiX, Hombrechtikon, Switzerland) according to the manufacturer´s instructions. Mouse tumor tissue was homogenized in a Tissue Lyser II (Qiagen, Hilden, Germany) und subsequently total RNA was isolated using the Nucleo Spin II kit, (Macherey and Nagel, Düren, Germany) according to the instructions of the manufacturers. The RNA was quantified by means of photometric measurement using the NanoDrop^®^-ND-1000 spectrophotometer (NanoDrop, Wilmington, DE, USA), and the integrity was proved by using the 2100 Bioanalyzer (Agilent Technologies, Palo Alto, CA, USA).

### Quantitative real-time PCR (qRT-PCR)

Experiments were performed as previosly described [37] with modifications. cDNA was produced from 0.5–1 μg total RNA with the SuperScript® III Platinum®Two Step qRT-PCR Kit (Life Technologies, Invitrogen) or the Transcriptor First Strand cDNA Synthesis Kit (Roche Diagnostics, Mannheim, Germany). TaqMan experiments were carried out on an ABI PRISM® 7000 or 7900HT Sequence Detection System using the FastStart Universal Probe Master (Rox) Kit (Roche Diagnostics) or the TaqMan® gene expression Assay (Life Technologies, Applied Biosystems, Carlsbad, CA) for human TAP1 (Hs00388682), IFN inducible genes IFI16 (Hs00194261_m1), IFI27 (Hs00271467_m1), IFIT2 (Hs00533665_m1), OAS1 (Hs00973637_m1) and MxA (myxovirus resistance gene A; Hs00895608_m1) according to the manufacturer’s recommendations. The probes Hs99999901 18SrRNA and Hs99999905 GAPDH were selected to detect human 18SrRNA and GAPDH mRNA as internal references to normalize the target transcript. TaqMan® gene expression Assay (Applied Biosystems, Carlsbad, CA) Mm00443188 TAP1 was selected to detect mouse TAP1 mRNA and the Mm99999915 probe for mouse GAPDH was used as internal reference.

### Peptide binding assay, peptide transport assay and detection of TAP subunits in THP1 cells

Preparation of crude membranes was performed with 2 x 10^8^ THP1 cells as described previously [38]. For peptide binding assays 150 μg total protein of TAP-containing crude membranes were incubated with 1 μM radioactive ^125^I labeled antigenic peptide RRYQKSTEL as described before [38]. Bound peptide was separated by filter assay and quantified by γ-counting. For specific binding, background binding, determined in the presence of 450-fold excess of the viral TAP inhibitor ICP47, was subtracted. Peptide transport assay was done with 200 μg total protein of crude membranes and 1 μM nonameric peptide RRYQNSTC(ϕ)L (ϕ resembling iodoacetamido fluorescein coupled to cysteine) as described in Herget et al. [38]. After termination of the transport assay N-core-glycosylated and thus transported peptides were purified and quantified with a fluorescence plate reader (λ_ex/em_ = 485/520 nm; Polarstar Galaxy, BMG Labtech, Offenburg, Germany). For immunoblot detection of TAP subunits crude membranes (100 μg total protein) were solubilized with SDS-sample buffer. Proteins were analyzed by 10% SDS-PAGE followed by immunoblotting using the monoclonal antibody 148.3 against TAP1 [32,33]. After incubation with anti-mouse-HRP conjugated antibody, TAP1 was visualized by chemiluminescence using a Lumi Imager F1 (Roche Diagnostics, Mannheim, Germany).

### Murine model for malignant melanoma

All animal experiments in this study were performed complying with the German Animal Protection Law following procedures reviewed and approved by institutional and state animal protection boards. Ten-week-old male C57BL/6 mice (The Jackson Laboratory, Bar Harbor, ME) were anesthetized and inoculated subcutaneously above the caudal vertebra with 2.5x10^5^ B16F1 malignant melanoma cells (day 0). The mice received either no treatment (control group) or recombinant murine IFNα. IFNα treatment (10,000 IU) started on day +3 after application of B16F1 cells for 5 consecutive days and was administered by subcutaneous injection into the same region, where the B16F1 cells had been injected before. Measurement of tumor growth started three days after application of the first IFNα injection on day +6. Tumor size was callipered daily for 6 days until day +11 and tumor volume was calculated according to v = 1/2A x B^2^, where v is the volume, A denotes the largest dimension of the tumor and B represents the smallest dimension [39]. On day +7.5 and thus 12 hours after the last IFNα application three IFNα treated and three control mice were sacrificed and blood was collected by cardiac puncture for qRT-PCR analysis of TAP1 expression. Three days after the last injection of IFNα (day +11), three mice of the IFNα treated as well as three mice of the control group were sacrificed and tumors were excised for qRT-PCR analysis of TAP1 expression.

### Statistical analysis

Statistical analysis was performed with Sigmaplot Version 11 using Student’s t-test. Values of p<0.05 = *; p<0.01 = **; p<0.001 = *** were considered as significant and are indicated in the figures.

## Results

### IFNα-2b (IntronA) upregulates TAP1 expression in PBMCs of patients with malignant melanoma receiving adjuvant high-dose immunotherapy

**Fig 1:**
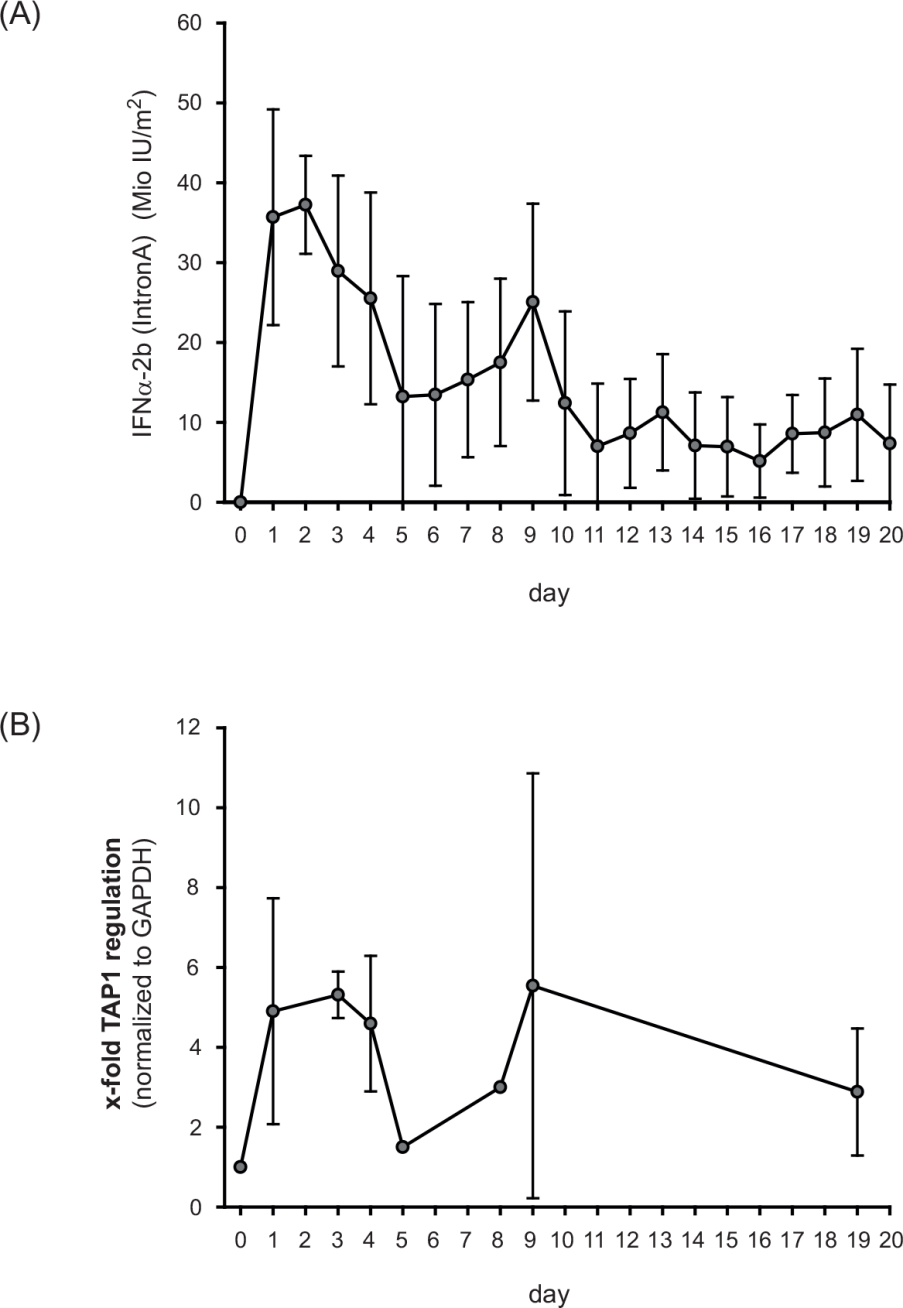
IFNα-2b (IntronA) stimulates TAP1 expression in peripheral blood mononuclear cells (PBMC) of patients (n = 18) with malignant melanoma receiving adjuvant high-dose immunotherapy. (A) The actual administered dose of IFNα-2b was about 20 million IU/m^2^ dependent on the clinically observed side effects. Only days of IFNα treatment are shown. (B) mRNA expression of TAP1 in PBMCs of patients treated with adjuvant high-dose IFNα-2b. Statistical analysis of TAP expression was performed using the *Statistical Analysis System* of the SAS Institute Inc. (Cary, NC, USA).

To study the mechanisms of action of IFNα-2b (IntronA) in adjuvant immunotherapy of metastatic melanoma we investigated the expression of TAP1 in peripheral blood mononuclear cells (PBMCs) of 18 patients diagnosed with UICC stage III malignant melanoma. Clinical characteristics of the study population are described in Table 1. The actual administered dose of IFNα was about 20 million IU/m^2^ dependent on the clinically observed side effects of IFNα (Fig 1A). As demonstrated in Fig 1B, IFNα treatment led to an upregulation of TAP1 expression in PBMCs of patients with malignant melanoma. The dimension of this stimulatory effect was dependent on the dose of the applied IFNα. This becomes apparent in the parallel run of the curves of the administered IFNα (Fig 1A) and the determined TAP1 expression (Fig 1B). Individual TAP1 regulation of each patient receiving high-dose interferon therapy is shown in S1 Fig in the supplement. The statistical analysis of the data using the *Statistical Analysis System* of the SAS Institute Inc. (Cary, NC, USA) resulted in an average 4-fold enhancement of TAP1 expression in comparison to TAP1 mRNA levels in PBMCs before therapy.

**Table 1:** Clinical characteristics of the patients.

patients (n)	age (years) of initial diagnosis	sex	localisation of primary tumour	tumour thickness (mm)	initial tumour stage	TNM	clark level	S100 value	SNB	SNB diagnosis	SNB localisation	lymph-adenectomy	localisation lymphadenectomy	side effects of IFNα therapy	5-years survival after therapy
1	28	M	malleolus medialis right	3,20	IIIA	pT4aN1aM0	4	0,05	yes	positive	inguinal right	yes/negative	inguinal right	fever; arthralgia; leukopenia	yes
2	34	M	digitus pedis IV left	3,50	IIIA	pT4bN1aM0	4	0,05	yes	positive	inguinal left	yes/negative	inguinal left	fever; arthralgia; leukopenia	yes
3	59	M	unknown primarius	-	IIIB	TxN1bM0	-	0,03	no	-	-	yes/positive	axillary right	fever; arthralgia; leukopenia	yes
4	62	M	unknown primarius	-	IIIB	TxN1bM0	-	0,00	no	-	-	yes/positive	supraclavicular right	fever; arthralgia; vertigo; nausea; leukopenia	no
5	66	M	back left	0,70	IIIB	pT1bN2aM0	4	0,08	yes	positive	axillary left	yes/positive	axillary left	arthralgia; leukopenia	no
6	55	M	foot sole left	7,00	IIIB	pT4bN2cM0	4	0,12	yes	positive	inguinal left	yes/positive	inguinal left	fever; headache; myalgia; leukopenia	yes
7	65	M	scapula right	7,40	IIIB	pT4bN2aM0	4	0,07	yes	positive	axillary right	yes/positive	axillary right	headache; leukopenia	no
8	45	M	thoracal right	0,60	IIIB	pT1aN2aM0	4	0,07	no	-	-	yes/positive	axillary right	fever; headache; leukopenia	no
9	56	M	submammary left	3,40	IIIA	pT3aN1aM0	4	0,05	yes	positive	axillary left	yes/negative	axillary left	headache; leukopenia	yes
10	45	M	thoracic spine	2,20	IIIA	pT3aN1aM0	4	0,05	yes	positive	inguinal left	no	-	headache; leukopenia	yes
11	38	M	femur right	1,62	IIIA	pT2aN1aM0	4	0,08	yes	positive	inguinal right	yes/negative	inguinal right	fever; arthralgia; leukopenia	yes
12	30	M	scapula left	6,00	IIIC	pT4aN3aM0	4	0,03	yes	positive	axillary left	yes/positive	axillary left	fever; arthralgia; myalgia; leukopenia	yes
13	52	M	regio retroauricularis left	6,00	IIIA	pT4aN1aM0	4	0,41	no	-	-	yes/positive	cervical left	headache; arthralgia; leukopenia	no
14	44	M	scapula right	5,53	IIIA	pT4aN1aM0	4	0,08	yes	positive	axillary right	yes/negative	axillary right	arthralgia; myalgia; leukopenia	yes
15	39	M	crus left	1,80	IIIA	pT2aN1aM0	4	0,10	yes	positive	inguinal left	yes/negative	inguinal left	fever; arthralgia; myalgia; leukopenia	yes
16	64	M	Back	2,00	IIIA	pT2aN1aM0	4	0,10	yes	positive	axillary left	yes/negative	axillary left	fever; arthralgia; myalgia; leukopenia	yes
17	42	M	femur right	1,00	IIIA	pT2aN1aM0	2	0,05	yes	positive	inguinal right	yes/negative	inguinal right	headache	no
18	46	M	foot sole right	4,00	IIIA	pT3aN1aM0	2	n.d.	yes	negative	inguinal right	no	-	fever; arthralgia; myalgia; leukopenia	no

Legend: male (m), SNB (sentinel lymph node biopsy), n.d. (not done); leukocytes normal range (4300/μl– 10,000/μl)

### IFNα induces expression of TAP1 in mouse blood and tumor tissue and suppresses subcutaneous melanoma metastasis in an in vivo tumor model

**Fig 2:**
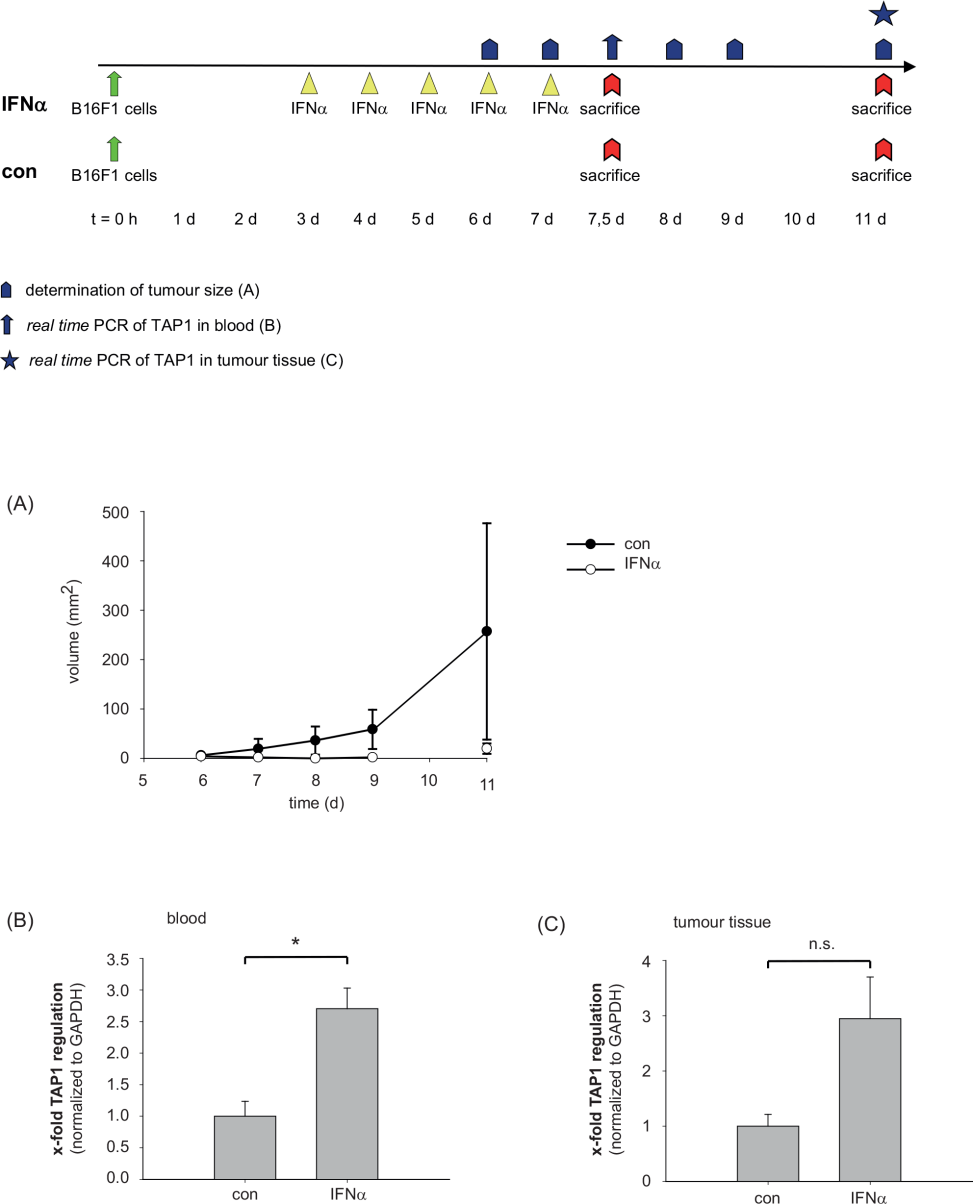
IFNα stimulates TAP1 expression in mouse blood and tumor tissue and suppresses subcutaneous melanoma metastasis in a murine model for malignant melanoma. C57BL/6 mice were inoculated subcutaneously with B16F1 malignant melanoma cells (day 0). The mice received either no treatment (control group, n = 3) or recombinant murine IFNα (n = 3). IFNα treatment (10.000 IU) started on day +3 after application of B16F1 cells for 5 consecutive days. (A) Measurement of tumor growth in control mice and IFNα treated mice. (B) On day +7.5, three IFNα treated and three control mice were sacrificed and blood was collected for qRT-PCR analysis of TAP1 expression. (C) Three days after the last injection of IFNα (day +11), three mice of the IFNα treated and three mice of the control group were sacrificed and tumors were excised for qRT-PCR analysis of TAP1 expression. Statistical analysis was performed using unpaired Student’s t-test (* p<0.05; not significant (n.s.)).

C57BL/6 mice were inoculated subcutaneously above the caudal vertebra with B16F1 malignant melanoma cells (day 0). The mice received either no treatment (control group) or adjuvant recombinant murine IFNα (10,000 IU). While the tumor volume of control mice constantly increased from day 6 to day 11 there were no visible tumors in IFNα-treated animals at day 6, 7, 8, and 9 and only small tumors at day 11 (Fig 2A). On day +7.5 and thus 12 hours after the final IFNα application, blood of three IFNα-treated and three control mice was collected for qRT-PCR analysis of TAP1 expression. As shown in Fig 2B, IFNα treatment enhanced significantly the TAP1 expression in peripheral blood (p = 0.013). To test whether this stimulatory activity of IFNα is also seen in the tumor tissue itself we excised tumors from IFNα-treated and control mice three days after the final injection of IFNα (day +11) and analyzed TAP1 mRNA by qRT-PCR in tumor tissue (Fig 2C). In the minimal tumor tissue present in IFNα-treated mice up to 5-fold higher TAP1 mRNA levels could be detected in comparison to TAP1 mRNA levels in tumors of the untreated control group (p = 0.068). In summary, a correlation was observed between an enhanced TAP1 mRNA expression in peripheral blood (Fig 2B) and tumor tissue (Fig 2C) and the suppressed formation of subcutaneous melanoma metastasis (Fig 2A) in animals receiving adjuvant IFNα therapy.

### IFNα-induced TAP1 expression in human THP1 and A375 cells

After demonstrating the stimulatory effect of IFNα on expression of TAP1 in primary human monocytic cells, murine blood cells and melanoma tissue we investigated the effect of IFNα on the expression of TAP1 *in vitro* in monocytic THP1 cells and the melanoma cell line A375.

**Fig 3:**
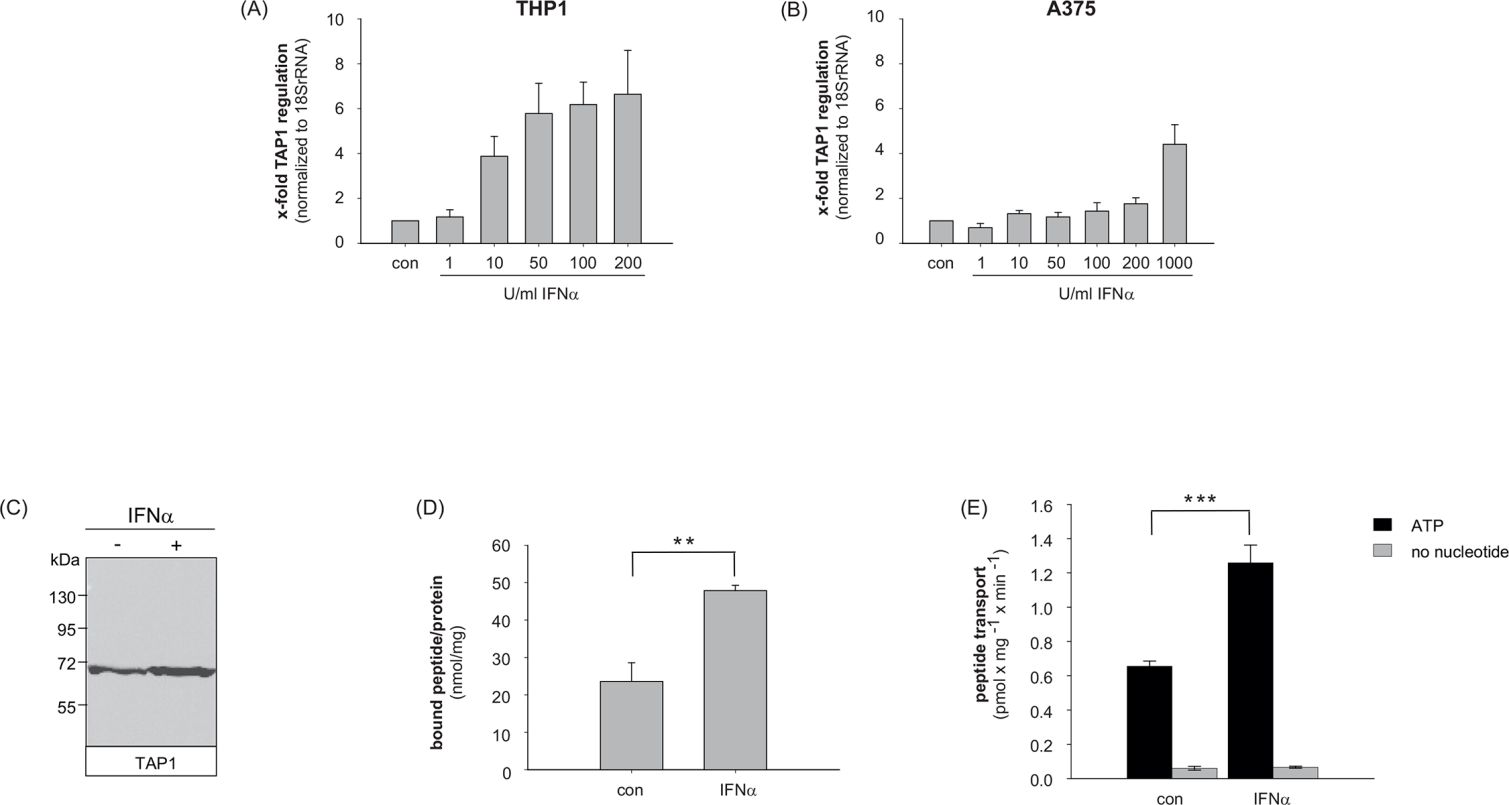
Stimulatory effects of IFNα on TAP expression, peptide binding and transport. THP1 cells and A375 cells were treated with the indicated concentrations of IFNα for 3 hours (A, B). After stimulation, TAP1 mRNA and 18SrRNA mRNA (as internal reference) were measured by qRT-PCR analysis. Depicted are -fold changes relative to untreated cells = standard error of mean (SEM) of three independent experiments. (C) TAP1 protein expression is induced by IFNα in THP1 cells measured by western blot analysis. (D) TAP dependent peptide-binding sites are increased significantly by IFNα in THP1 cells (p = 0.0072). (E) ATP-dependent peptide transport is stimulated significantly by IFNα in THP1 cells (p = 0.0006). Statistical analysis was performed using unpaired Student’s t-test (** p<0.01, *** p<0.001).

Therefore, human THP1 (Fig 3A) and A375 (Fig 3B) cells were stimulated with indicated concentrations of IFNα or left untreated. Whereas in A375 cells doses up to 1000 U/ml IFNα are needed to induce a significant TAP1 expression of approximately 4-fold (Fig 3B) much lower doses of IFNα are sufficient to enhance the transcription in THP1 cells. As shown in Fig 3A, already 10 U/ml IFNα increase TAP1 expression 4-fold in comparison to untreated THP1 control cells. A maximum of TAP1 induction in THP1 cells was seen after stimulation with 200 U/ml IFNα. Initial time course experiments indicated that the stimulatory effect of IFNα increases up to 6 h of treatment, then declines. However, enhanced levels of TAP1 expression could be observed for at least 48 h in THP1 and A375 cells (data not shown). Furthermore, IFNα-dependent TAP1 expression was compared to the regulation of IFN inducible genes IFI16, IFI27, IFIT2, OAS1 and MxA (myxovirus resistance gene A). Besides TAP upregulation, we also observed an upregulation of these genes in the cell lines A375 and THP1 treated with different IFNα concentrations (S2 Fig in the supplement).

### TAP expression, TAP binding affinity and TAP transport activity is induced by IFNα in THP1 cells

We next focused on the function of the TAP protein and examined whether the transport activity is enhanced in IFNα-stimulated THP1 cells in comparison to untreated control cells. Therefore, THP1 cells were stimulated with 200 U/ml human IFNα for 6 h. As shown in Fig 3C, an IFNα-induced enhancement of TAP1 protein amounts was observed. We next analyzed the TAP binding affinity (Fig 3D) in TAP containing crude membranes of THP1 cells. A significant 2-fold stimulation of peptide binding was observed in the presence of IFNα (p = 0.0072). Furthermore, we could show that TAP containing membranes show an ATP-dependent peptide transport activity. The transport strictly requires ATP hydrolysis since no peptide translocation by TAP is observed in the absence of ATP. Peptide transport is doubled after stimulation of the cells with IFNα (Fig 3E; p = 0.0006). In summary, coupling between TAP binding activity and transport activity could be shown. Both processes occur twice as effective upon IFNα stimulation.

### IFNα mediates phosphorylation of STAT1 and STAT3 in THP1 and A375 cells

To study the molecular mechanisms involved in the IFNα-induced TAP1 expression, THP1 and A375 cells were stimulated with increasing concentrations of IFNα as indicated. Subsequently, activation pattern of several signalling pathways such as JAK/STAT, MAP kinases ERK1/2, and protein kinase B/Akt was analyzed by Western blot analysis.

**Fig 4:**
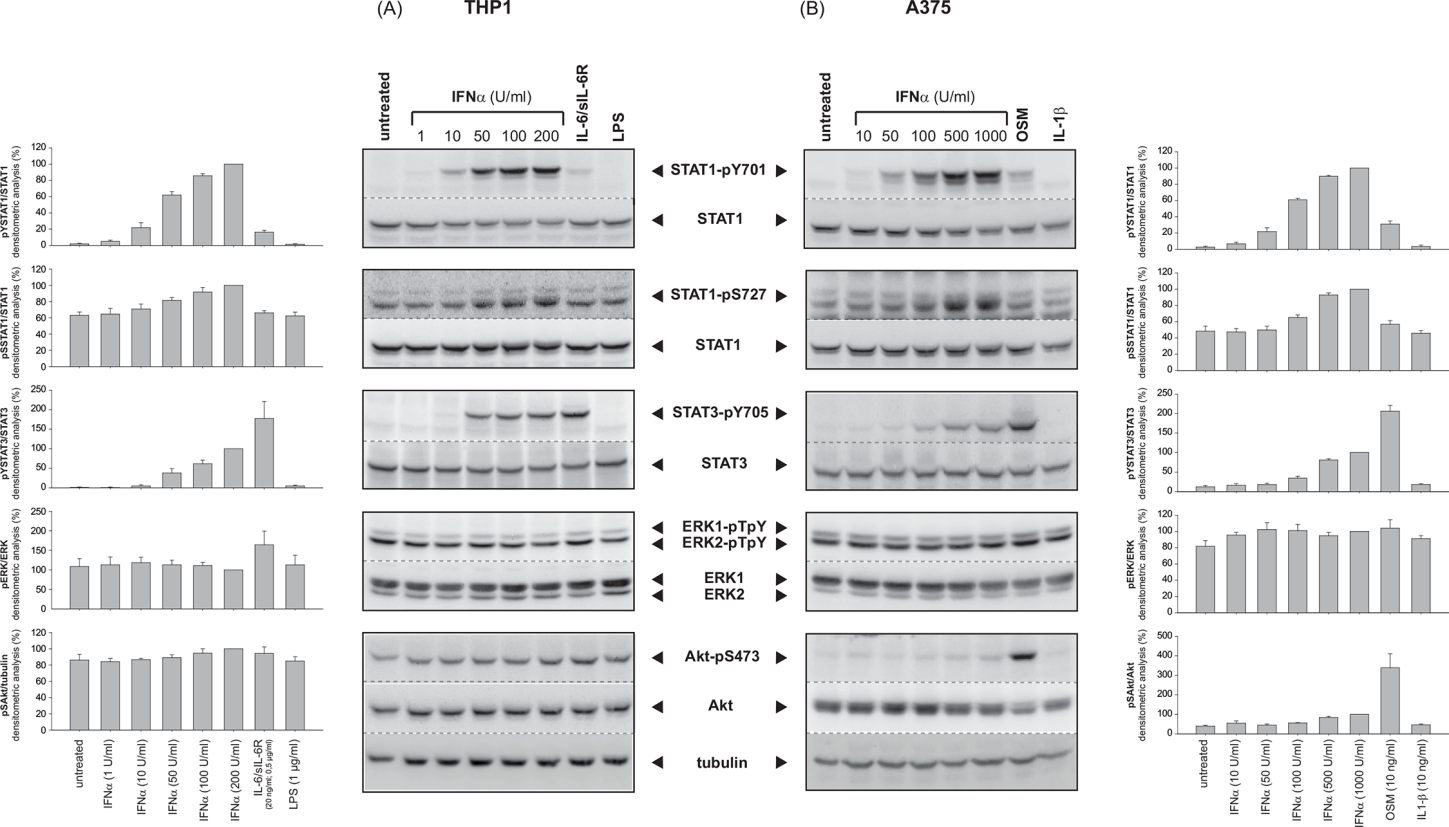
IFNα-activated signalling pathways in THP1 and A375 cells. THP1 (A) and A375 (B) cells were stimulated with the indicated concentrations of IFNα. As controls, THP-1 cells were additionally stimulated with IL-6/sIL-6R and LPS; A375 cells were stimulated with OSM or IL-1β. The phosphorylation levels of STAT1, STAT3, ERK1/2 and Akt were detected via Western blot analysis. Displayed are mean values of at least three independent experiments.

In THP1 cells, a statistically significant STAT1 tyrosine phosphorylation was observed already at a dose of 50 U/ml IFNα, while for significant serine phosphorylation of STAT1 200 U/ml IFNα are required (Fig 4A, 1^st^ and 2^nd^ panel, lanes 2–6). In contrast, in A375 cells 100 U/ml IFNα are needed to induce a significant STAT1 tyrosine phosphorylation and only at concentrations beyond 500 U/ml IFNα the serine residue 727 in STAT1 is significantly phosphorylated (Fig 4B, 1^st^ and 2^nd^ panel, lanes 2–6). Furthermore, increasing concentrations of IFNα also lead to activation of the transcription factor STAT3 in both cell types (Fig 4A and 4B, 3^rd^ panel, lanes 2–6), but in comparison to IFNα-mediated STAT1 activation to a lesser extent. In order to clarify whether IFNα has only a weak potential to activate STAT3, THP1 and A375 cells were stimulated with interleukin-6 (IL-6)-type cytokines (THP1: IL-6/sIL-6R; A375: Oncostatin M, OSM), which are well-known activators of STAT3. Indeed, for IL-6/sIL-6R (THP1) and OSM (A375) a reciprocal situation is observed: the tyrosine phosphorylation of STAT3 is very strong (Fig 4A and 4B, 3^rd^ panel, lane 7) while only a weak tyrosine phosphorylation and interestingly no serine phosphorylation of STAT1 is detectable (Fig 4A and 4B, 1^st^ and 2^nd^ panel, lane 7). In contrast, in this setting, threonine/tyrosine phosphorylation of ERK1/2 by IFNα treatment could neither be observed in THP1 nor in A375 cells (Fig 4 A and 4B, 4^th^ panel). We could show that both cell types displayed a high basal ERK1/2 phosphorylation, which was not further increased by IFNα treatment. Furthermore, a weak, but significant increase in the serine phosphorylation of Akt was observed upon IFNα treatment in A375 cells (Fig 4B, 5^th^ panel), which is much more prominent upon stimulation with OSM. In THP1 cells, which display a high basal serine phosphorylation level of Akt (S473), IFNα was without any further effect (Fig 4A, 5^th^ panel).

Taken together, these data show that IFNα preferentially activates STAT1 over STAT3 in THP1 and A375 cells. Only minor activation of the PKB/Akt pathway could be observed and no further stimulation of the pre-activated MAPK ERK1/2 was detectable.

### The inhibition of Janus kinases abrogates the IFNα-induced TAP1 expression in THP1 and A375 cells

**Fig 5:**
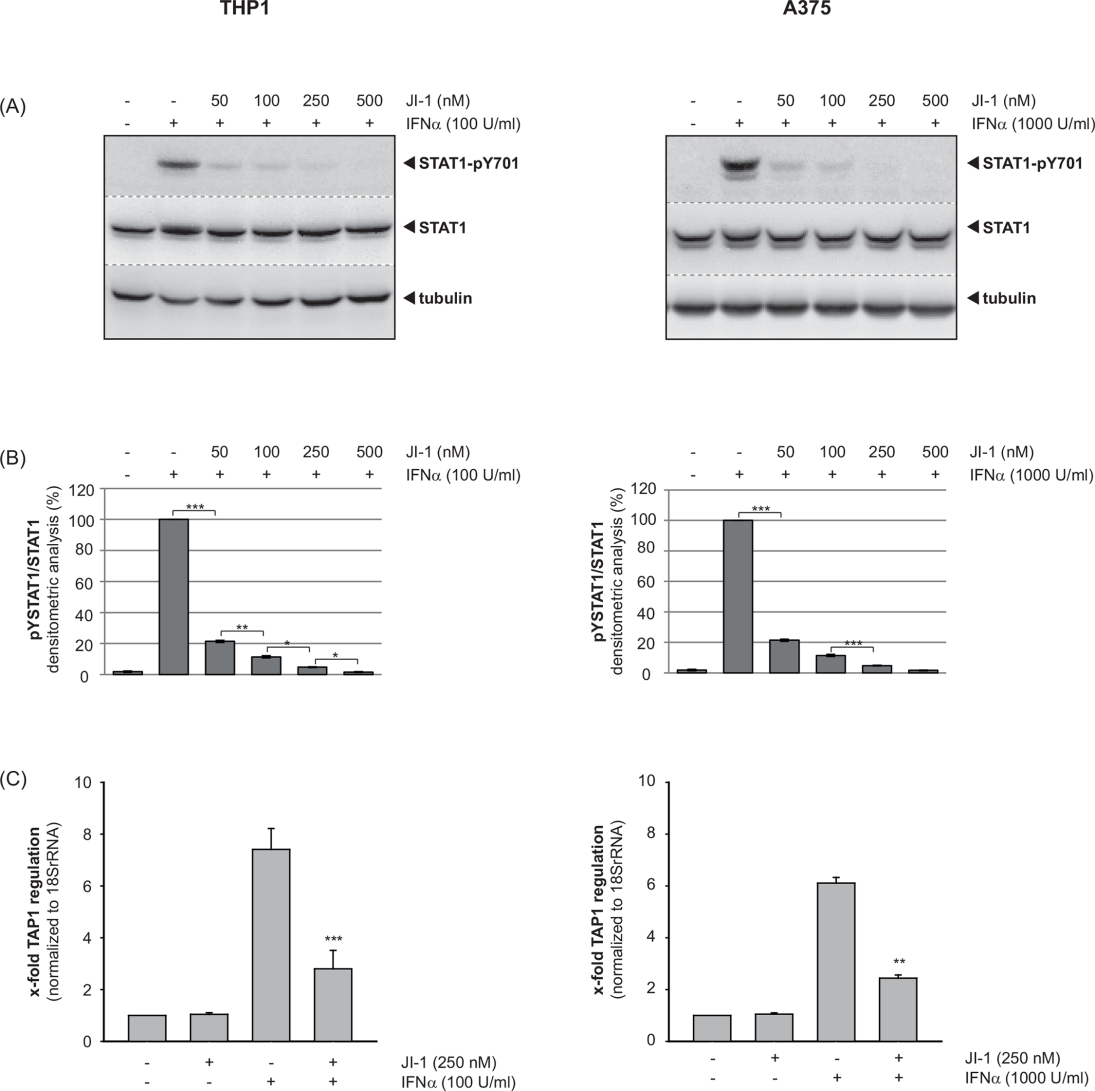
The inhibition of Janus kinases abrogates the IFNα-upregulated TAP1 expression in THP1 and A375 cells. (A) THP1 (left) or A375 (right) cells were pre-incubated with the indicated amounts of JAK inhibitor I (JI-1), followed by stimulation with 100 U/ml or 1000 U/ml IFNα, respectively. Western blot analysis was performed using a specific antibody against phosphorylated STAT1 (STAT1-pY701), a STAT1 antibody recognizing the protein irrespective of its phosphorylation status and with an α-tubulin antibody. (B) Phosphorylation intensities were quantified by chemiluminescence analysis and normalization to loading controls. Shown are the means (n = 3) with standard error of mean (SEM, two-tailed, paired Student’s t-test, * p<0.05, ** p<0.01, *** p<0.001). (C) THP1 (left) or A375 (right) cells were pretreated with JI-1, and subsequently exposed to 100 U/ml or 1000 U/ml IFNα. Relative expression levels of TAP1 were analyzed by qRT-PCR and normalized to 18SrRNA as internal reference. Shown are -fold changes relative to unstimulated control = SEM (n = 3). Statistical significance was evaluated by performing a two-tailed, paired Student’s t-test (** p<0.01, *** p<0.001).

To clarify the molecular mechanisms underlying the specific increase in TAP1 mRNA expression through IFNα stimulation in more detail, we pre-incubated THP1 (A) and A375 (B) cells with the JAK inhibitor I (JI-1) for 15 min, followed by stimulation with IFNα for 15 min. Whole cell lysates were subjected to Western blot analysis using a specific antibody against tyrosine-phosphorylated STAT1 (pY-STAT1). Pre-incubation of the cells with JI-1 significantly reduced IFNα-induced STAT1 tyrosine phosphorylation in a concentration dependent manner in both cell types (Fig 5A and 5B). Furthermore, total RNA was prepared 6 h after IFNα treatment and relative expression levels of TAP1 were analyzed by qRT-PCR. TAP1 mRNA was strongly induced by IFNα treatment as observed before. However, the presence JI-1 strongly suppressed the IFNα-induced TAP1 gene expression by nearly 60% in THP1 and A375 cells (Fig 5C).

## Discussion

Survival of vertebrates is strongly dependent on the challenging task of the adaptive immune system to protect the organism against invaders and cancer. The antigen presentation machinery by major histocompatibility complex (MHC) class I molecules is imperative to display intracellular antigens for the generation and maintenance of CD8^+^ T-cell responses to malignantly transformed cells. The transporters associated with antigen processing (TAP1, TAP2) play a pivotal role in the MHC class I dependent immune surveillance by mediating the transport of antigenic peptides from the cytoplasm to the ER lumen for loading onto MHC class I molecules and subsequent delivery to the cell surface through the secretory pathway. Aberrant expression or function of TAP facilitates tumor cells, including melanoma cells, to escape the immune surveillance by suppression of the antigen processing and cell surface presentation of tumor specific peptides via MHC class I molecules which is associated with an ineffective T-cell response [24,25].

In previous work, downregulation of TAP1 protein expression could be observed in primary melanoma lesions that is associated with significant higher invasive melanoma growth and a lack of tumor-infiltrating lymphocytes [40]. Low TAP1 expression correlates with tumor progression, loss of regression and increased development of melanoma metastases [40–42]. Furthermore, clinical investigations showed that reduced expression of TAP1 represents an independent prognostic marker for melanoma [41,43].

In this study, we showed that adjuvant IFNα therapy clearly increases TAP1 expression in PBMCs of stage III melanoma patients. To gain more insight into the biological effects of IFNα on TAP1 regulation, we investigated the *in vivo* anti-tumor activity of this molecule against murine melanoma cells using the murine C57BL/6-B16F1 tumor transplantation model. Earlier studies demonstrated that B16F1 cells cultivated *in vitro* and then transplanted subcutaneously into C57BL/6 mice produced an experimental model of tumor growth and vascularization with characteristics similar to the initial tumor [44]. Using this model, a clear correlation of exogenously administered IFNα on the proliferative potential of transplanted B16 melanomas and enhanced TAP1 expression in peripheral blood and tumor tissue was demonstrated. Accordingly, IFNα treatment led to a dose-dependent increase in TAP1 transcription as well as an increased TAP transport and binding affinity in the monocytic cell line THP. A possible explanation for the strong anti-tumorigenic effect of IFNα could therefore be the enhanced cell surface presentation of tumor specific peptides via MHC class I molecules as a result from upregulation of TAP1 expression. Consequently, an enhanced activation of cytotoxic T-cells and a cancer-specific immune response should be envisaged in IFNα responsive patients.

It is known that only a subgroup of patients benefits from adjuvant IFNα treatment measured by significant higher recurrence-free survival [4]. A possible explanation could be a higher IFNα-induced expression of TAP1 in this patient group. Indeed, low TAP1 expression or loss of TAP transporter function in melanoma allows evasion from immune surveillance through a lack of tumor antigen presentation [23]. To date, different groups investigated the impact of TAP1 on MHC I antigen presentation in tumor cells and addressed TAP1 downregulation in melanoma as a tumor-specific immune escape mechanism. It was shown that transfection or adenoviral inoculation of TAP1 in melanoma cells increase tumor-specific immune response *in vitro* and *in vivo* and restore immune surveillance against melanoma [30,45,46].

It seems reasonable to suppose that TAP1 downregulation provides tumor cells with a mechanism to escape cytotoxic T-lymphocyte recognition explaining ineffectiveness of specific vaccination strategies. The observed benefit of IFNα treatment could be mediated by the shown dual effect of increased TAP1 expression on the one hand in antigen presenting cells and on the other hand in ‘silent’ metastatic melanoma cells. A combination of IFNα with a specific target-oriented immunotherapy in metastatic melanoma patients could be an approach to increase the anti-tumor response.

Because peptide binding, transport and ATP hydrolysis are tightly coordinated in the antigen transport complex TAP, we wanted to know how IFNα affects the mentioned functions of this transport protein in antigen presenting cells. Our data revealed that TAP expression, TAP binding affinity and TAP transport activity is induced by IFNα in THP1 cells. Subsequently, we analyzed the molecular mechanisms involved in the IFNα-induced TAP1 expression. Our data show that IFNα clearly activates phosphorylation of STAT1 and STAT3 but preferentially activates STAT1 over STAT3 in THP1 and A375 cells. Inhibition of Janus kinases abrogates the IFNα-induced TAP1 expression in these cell lines. These results emphasize that the JAK/STAT pathway is a crucial mediator for TAP1 expression elicited by IFNα treatment, as analyzed in detail before [47]. However, a smaller study provided evidence that STAT1 expression in melanoma tissue does not correlate with patients’ response to adjuvant IFNα [12]. Interestingly, Wang et al. (2007, 2008) showed that the balance of pSTAT1/pSTAT3 is modulated in human melanoma lymph node metastasis of patients receiving high-dose IFNα in a neoadjuvant setting [48,49]. High-dose IFNα therapy upregulated pSTAT1 expression and downregulated pSTAT3 expression and MEK/ERK MAPK pathway in human melanoma lymph node metastasis in immunhistological analysis [48,49]. Another important finding was that STAT3 activation appeared not be associated with the MEK/ERK MAPK pathway in human melanoma *in vivo* [49]. Also TAP2 was upregulated in human melanoma cells after IFNα therapy [48]. In contrast to our current and previous work [28], which showed an upregulation of TAP1 in PBMCs of IFNα treated patients, they found no TAP1 regulation in tissue of human melanoma lymph node metastasis after IFNα treatment [48].

Furthermore, our *in vitro* analyses revealed a phosphorylation of STAT3 in the A375 melanoma cell line. Activation of this transcription factor has been linked to abnormal cell growth and transformation as well as to IFNα-mediated growth suppression *in vitro* [50]. Interestingly, Humpoliková-Adámková et al. (2009) found a significant correlation between the length of survival end-points and a lack of STAT3 activation in examined primary cultures established from node metastases of melanoma patients that were monitored in a 5-year clinical follow-up [51]. They showed IFNα-stimulated STAT3 phosphorylation at tyrosine (Y705) residue in 17% of cases. These over-reactive cell populations originated from patients who had the shortest disease-free intervals [51]. Another important point worth mentioning is the presence of gene polymorphisms in the JAK/STAT pathway. For example, STAT3 polymorphism predicts IFNα response in patients with other tumor entities like renal cell carcinoma [52] or chronic myeloid leukemia [53]. Taken together, the downstream JAK/STAT pathway is essential in mediating IFNα-induced effects in melanoma.

A cancer biomarker can be defined as a biological feature that provides diagnostic, prognostic, predictive, or therapeutic information about a particular disease or subject [54]. In conclusion, the search for melanoma biomarkers is a very important on-going process [29] especially in the context of personalized medicine. At present such a gold-standard prognostic or predictive marker for therapy response is unfortunately not available for malignant melanoma. Understanding the molecular mechanism of IFNα effects and prediction of response in the IFNα therapy regime allows initiation and continuation of IFNα treatment for responders and exclusion of non-responders to avoid therapy inefficacy and side-effects. Combination of IFNα with additional vaccination strategies for patients with high IFNα-induced TAP1 expression in PBMCs could be regarded as an option for metastatic melanoma patients. Although the investigated collective is small, we pursue a long-time follow up giving us more information about relevance of TAP1 for IFNα therapy response and its impact on prognosis.

## Supporting Information

S1 Fig Individual TAP1 regulation of each patient receiving high-dose interferon therapy. Black lines depict relative TAP1 mRNA regulation (y-axis on the right), the grey bars show the administered dose of IFNα on the corresponding day of treatment (y-axis on the left).
(EPS)

S2 Fig Regulation of other INFα induced genes compared to TAP1 expression. Cells were stimulated with the indicated concentrations of IFNα. Afterwards, real-time PCR analysis of the IFN inducible genes IFI16, IFI27, IFIT2, OAS1 and MxA (myxovirus resistance gene A) was performed.
(EPS)
